# RNAseq Transcriptional Profiling following Whip Development in Sugarcane Smut Disease

**DOI:** 10.1371/journal.pone.0162237

**Published:** 2016-09-01

**Authors:** Patricia D. C. Schaker, Alessandra C. Palhares, Lucas M. Taniguti, Leila P. Peters, Silvana Creste, Karen S. Aitken, Marie-Anne Van Sluys, João P. Kitajima, Maria L. C. Vieira, Claudia B. Monteiro-Vitorello

**Affiliations:** 1 Departamento de Genética, Universidade de São Paulo, Escola Superior de Agricultura “Luiz de Queiroz”, Piracicaba, São Paulo, Brazil; 2 Instituto Agronômico de Campinas, Centro de Cana, Ribeirão Preto, São Paulo, Brazil; 3 CSIRO Agriculture, Queensland Bioscience Precinct, St Lucia, Queensland, Australia; 4 Departamento de Botânica, Instituto de Biociências, Universidade de São Paulo, São Paulo, São Paulo, Brazil; 5 Mendelics Análise Genômica, São Paulo, São Paulo, Brazil; University of California Riverside, UNITED STATES

## Abstract

Sugarcane smut disease is caused by the biotrophic fungus *Sporisorium scitamineum*. The disease is characterized by the development of a whip-like structure from the primary meristems, where billions of teliospores are produced. Sugarcane smut also causes tillering and low sucrose and high fiber contents, reducing cane productivity. We investigated the biological events contributing to disease symptoms in a smut intermediate-resistant sugarcane genotype by examining the transcriptional profiles (RNAseq) shortly after inoculating the plants and immediately after whip emission. The overall picture of disease progression suggests that premature transcriptional reprogramming of the shoot meristem functions continues until the emergence of the whip. The guidance of this altered pattern is potentially primarily related to auxin mobilization in addition to the involvement of other hormonal imbalances. The consequences associated with whip emission are the modulation of typical meristematic functions toward reproductive organ differentiation, requiring strong changes in carbon partitioning and energy production. These changes include the overexpression of genes coding for invertases and trehalose-6P synthase, as well as other enzymes from key metabolic pathways, such as from lignin biosynthesis. This is the first report describing changes in the transcriptional profiles following whip development, providing a hypothetical model and candidate genes to further study sugarcane smut disease progression.

## Introduction

**Fig 1:**
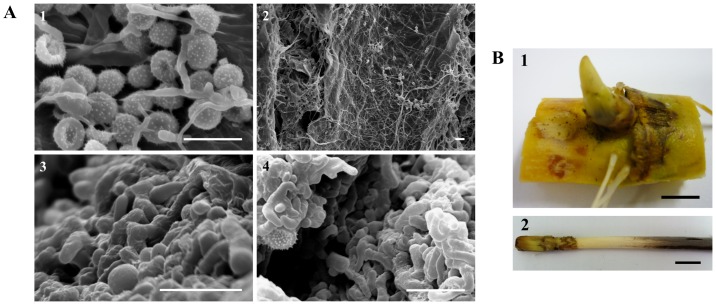
Sugarcane smut. **(A)** Scan electron microscopy of *S*. *scitamineum* hyphal growth in the sugarcane bud at 5 DAI (1, 2). Fungal sporogenesis and teliospore maturation in the base of the sugarcane whip at 200 DAI (3; 4). Bar = 10 μm. **(B)** (1) Sugarcane bud at 5 DAI. (2) Base of the whip at 200 DAI. Bar = 1 cm.

Sugarcane (*Saccharum* spp.)is the fifth most important crop in the world [1]. In addition to being a source of sugar for food, the crop has the potential to generate clean and renewable products, such as biofuels, bioplastics, bio-hydrocarbons, and bioelectricity. Due to its agronomic attributes, such as its high yield and ability to survive under adverse conditions [2], sugarcane is found in most of the 90 tropical and subtropical countries [1]. Nonetheless, the crop hosts several pathogens, including the fungus *Sporisorium scitamineum*, the causal agent of sugarcane smut disease [3] (Fig 1). Sugarcane smut is mainly characterized by the development of a long whip-like structure consisting of plant and fungal tissues in which billions of teliospores are produced. The name ‘smut’ derives from the black powdery mass of teliospores released by these structures that resemble soot. The whips originate in the primary meristems of the apex and lateral buds of infected stalks, and they are initially covered with a thin silvery membranous sheath [3], which detaches after the teliospores mature and are ready to disperse. In more susceptible varieties, whips can be detected as early as 2 to 4 months of age, with peak whip growth occurring in the 6th or 7th month [4]. Smut is mainly transmitted by wind-borne teliospores infecting standing canes but also by teliospores in the soil that infect planted setts. The germination of the teliospores leads to meiosis, which produces haploid sporidia. Mating-compatible sporidial cells produce infective hyphae through hyphal anastomosis, which initiates plant colonization [5].

The disease limits the crop yield and properties of sugarcane products, causing losses in cane tonnage and juice quality. Other disease symptoms include tillering and low sucrose and increased fiber contents [3]. Like most agronomic traits, smut resistance is a quantitative characteristic [6] that is difficult to genetically and functionally characterize. Moreover, modern varieties of sugarcane (2n = 100–130) have a complex genomic structure that derives from a highly polyploid and aneuploid interspecific hybridization [7, 8], hindering the understanding of the quantitative traits and mapping of their loci [9, 10]. Efforts to elucidate the molecular basis of sugarcane smut resistance have been made since James [11] proposed the existence of a chemical resistance mechanism. Lloyd and Pillay [12] identified some flavonoids, which are teliospore-germination inhibitors, and subsequently, a correlation between the resistance rating and concentration of glycosidic substances was established [13]. Later studies reported changes in the patterns of free polyamines and their conjugation in both susceptible and resistant sugarcane varieties infected by *S*. *scitamineum* [4,14]. Changes in the sugarcane gene expression profile induced by the fungus have been identified by several authors using techniques such as suppression-subtractive hybridization-based sequencing and differential display of complementary DNA-amplified fragment-length polymorphisms [15–18].

Despite these attainments, more detailed studies are needed to precisely define the changes in the entire sugarcane gene repertoire when challenged with the pathogen, both at different stages of fungal development and in different host tissues. Messenger RNA sequencing (RNAseq) technology has the potential to explore the complete set of gene expression programs to a high level of accuracy and depth, providing further insights into plant-pathogen interactions [19]. This method has been applied to several mixed-model systems of plant-fungus interactions [18,20–24], and more recently, to elucidate the early stages of the sugarcane-smut pathosystem [25–27]. Continuing the study of this pathosystem, we used RNAseq technology to perform a comparative analysis of infected sugarcane tissues of a smut intermediate-resistant genotype at two time points: shortly after inoculation and later, when the whips appeared and disease symptoms were evident (Fig 1). In addition to confirming the existing data [25], this work addresses the molecular events following whip emission. The most relevant conclusions are: 1) the association with transcriptional reprogramming of shoot apical functions probably by restraining floral development; 2) the transcriptional changes in carbon partitioning, mostly pronounced in hexoses and lignin; and 3) the relatedness of auxin to whip emission as well as the response associated with oxidative stress.

## Material and Methods

### Ethics Statement

*S*. *scitamineum* SSC39 teliospores were collected from experimental network areas of IAC sugarcane breeding program (Instituto Agronômico, Centro de Cana, Ribeirão Preto, São Paulo, Brazil), as described by Taniguti et. al (26). The healthy buds used to conduct the experiments were obtained from IAC sugarcane nursery. No special permits were necessary for teliospores and genotype used, because this project was developed in collaboration with IAC researchers. This work does not involve endangered or protected species.

### Experimental Design

*S*. *scitamineum* SSC39teliospores were checked for viability and were inoculated as previously described using artificial wounding method [26]. The initial sugarcane response was analyzed based on pools of 10 breaking buds collected at 5 DAI (days after inoculation). The late response was evaluated using culms after whips emerged at 200 DAI. Sampling was at the base of the whips, up to 2 cm below the culm. This is a region of intensive sugarcane cell division and fungal sporogenesis. Infected plants were compared with control (mock-inoculated) plants of the same age. Three biological replicates were included for each inoculated and control plant using a completely randomized design maintained on greenhouse benches (S1A File). A PCR amplicon containing the rDNA internal transcribed spacer region (ITS1, 5.8S and ITS2) of *S*. *scitamineum* generated with the primers Hs (5’ -AACACGGTTGCATCGGTTGGGTC- 3’) and Ha (5’ -GCTTCTTGCTCATCCTCACCACCAA- 3’) according to Bueno [28] was used to confirm infection at 5 DAI.

### RNA Extraction, Libraries, and Sequencing

Total RNA was extracted from the samples using distinct methods for each plant developmental stage as described by Taniguti *et al*. [26](S1A File). The quality of the total RNA was verified using an Agilent 2100 Bioanalyzer (Agilent Technologies, USA), and the libraries were constructed using a TruSeq RNA Sample Prep v2 Low Throughput (LT) kit as described in the manufacturer's instructions (Illumina, San Diego, CA). The libraries were paired-end sequenced using the Illumina system (HiScanSQ).

### Pre-Processing and Mapping the Illumina Reads

The Illumina reads were treated as previously described [26] (S1A File). Two reference sequences were used to map the RNAseq data: the complete genome sequence of *S*. *scitamineum* [26] and a set of unigenes produced by the assemblage of RNAseq data from six sugarcane cultivars [29]. The software packages used for mapping were Bowtie2 V2.1.0 [30] and BWA [31]. Bowtie2 was used with the default parameters in the sensitive mode (-D 15; -R 2; -L 22; -i S, 1, 1.15), while BWA alignments were obtained using the default parameters (-n 0.04; -k 2; -O 11). The RNAseq reads that showed no similarities to the sugarcane unigenes using the above parameters were assembled using Trinity [32]. Clusters identified by the prefix “gg” were then selected by comparison to the Viridiplantae sequences of UniProtKB [33].

### Sugarcane Gene Expression Analysis

The differentially expressed genes (DEGs) were identified using the DESeq2 package [34]. For the 5-DAI data, DEGs were considered to be statistically significant if they had a p-value less than 0.05 when compared with control buds. The multiple-test correction proposed by Benjamini and Hochberg [35] was used for the 200-DAI data by applying a FDR (False discovery rate) to generate a set of DEGs with the same significance level (<0.05). The DrawVenn webtool (http://bioinformatics.psb.ugent.be/webtools/Venn/) was used to produce Venn diagrams from the sets of DEGs obtained from the BWA/DESeq2 or Bowtie2/DESeq2 analyses as well as different reference sets.

### Annotation and Gene Ontology Analysis

The BLAST2GO tool V2.7.2 [36] was used with the default parameters to assign GO (Gene Ontology) terms to the DEGs. Metabolic pathways analysis was performed based on the KEGG database [37]. GO enrichment analysis was conducted with the BLAST2GO tool using the two-sided Fisher’s exact test with the p-value set at ≤ 0.05. The GenBank [38] and UniProt [33] databases and InterProScan [39], SignalP [40], TMHMM [41], ScanProsite, and MyDomains [42] tools were used to predict the function and features of the protein sequences.

### Quantitative PCR (qPCR) Expression Analysis

Quantitative PCR analysis was used to confirm the gene expression profiling data obtained from RNAseq. Transcripts encoding: invertase, auxin transporter, trehalose-6P synthase, pyruvate decarboxylase, aldolase, S-adenosylmethionine synthetase (SAM), peroxidase and longifolia-like protein (LGN) were selected for reverse transcription-qPCR (RT-qPCR) reactions (S2 File). The primers were manually designed and the quality verified using Gene Runner (http://www.generunner.net/) and NetPrimer (http://www.premierbiosoft.com/netprimer/). All RT-qPCRs were performed in a 7500 Fast Real-Time PCR System (Applied Biosystems,Waltham, MA) using a GoTaq^®^ One-Step RT-qPCR System Kit (Promega, Madison, WI). A reaction mixture containing 50 ng of RNA, 6.5 μL of GoTaq^®^ qPCRMaster Mix, 0.2 μM of each primer, 0.25 μL of GoScript^™^ RT Mix, and nuclease-free water to a final volume of 12.5 μL was used for the three biological replicates and two technical replicates. The cycling conditions were as follows: 37°C for 15 min; 95°C for 10 min; 40 cycles of 95°C for 10 s, 60°C for 30 s, and 72°C for 30 s. Primer specificity was confirmed by obtaining the dissociation curve for each reaction. Sugarcane housekeeping genes encoding polyubiquitin [43] and GAPDH (d-glyceraldehyde-3-phosphate dehydrogenase [44] were used to normalize the expression signals. The PCR efficiencies and Cq values were obtained using the LinReg PCR program [45]. Relative changes in the gene expression ratios were calculated using REST software [46]. Control samples (mock-inoculated plants) were used as calibrators. Student's*t*-test was used to estimate significant changes in the relative expression levels (p < 0.05).

## Results and Discussion

### General Analysis

In this study, the smut-intermediate resistant variety ‘RB925345’ developed whips and other disease symptoms beginning 127 days after inoculation (DAI). However, the plants were sampled at 200 DAI because this was the time when whips were detected in all three replicate plants used in the experimental design (S1A File). Of the total number of inoculated plants, 48 (53%) developed whip over the timeline of the experiment (334 days)(S1B File).

Samples of 5 DAI were used to amplify the 509-bp sequence that corresponds to the 5.8S ribosomal RNA gene and flanks internal transcribed spacers 1 and 2 in *S*. *scitamineum* [28], confirming the fungal infection (S1C File). It was particularly necessary to confirm infection 5 DAI, because the buds were collected with no smut disease symptoms.

A total of 225.2 million paired-end sequences (PEs) of ~100 bp (~22.5 Gbp) was obtained for the 12 RNAseq libraries (~18 million reads per library). Including the corresponding control libraries, 111,926,958 (49.7%) PEs were from the 5-DAI collection and 113,269,226 (50.3%) PEs were from the 200-DAI collection (S3A File). Fungal sequencing reads were screened after mapping them to the whole *S*. *scitamineum* SSC39B genome [26], leading to the removal of approximately 20% (2% 5 DAI; 18% 200 DAI) of the PEs (S3B File).

### Count-Based Differential Expression Analysis of the RNAseq Data

**Fig 2:**
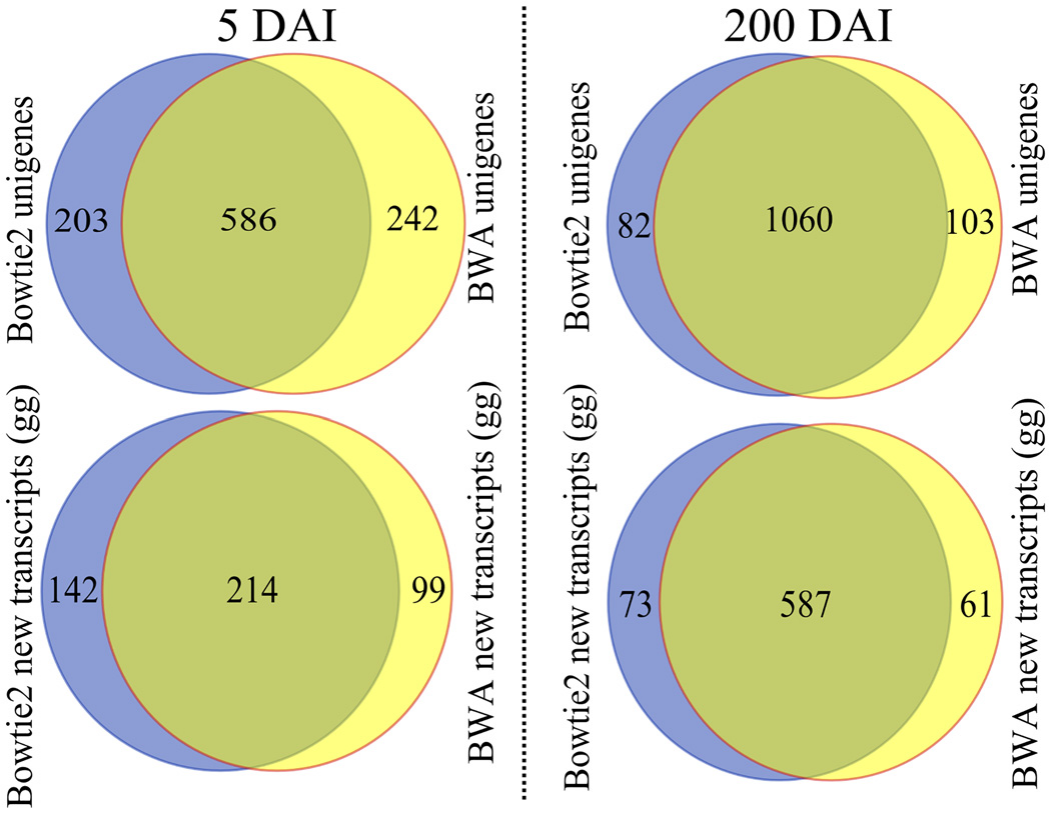
Differential expression gene analysis of 5-DAI and 200-DAI samples of the smut infected plants. Venn diagrams show the number of genes detected as differentially expressed (DEGs) by the DESeq2 package [33] using two mapping software systems: 1) Bowtie2 and 2) BWA, as well as two sets of references: 1) sugarcane unigenes [29]and 2) the novel sugarcane transcripts assembled from sugarcane unigenes unmapped sequences using Trinity [32]. Intersections of the identified DEGs from the mapping results were considered for the following analysis.

A set of previously obtained sugarcane transcripts was used to describe the biological events underlying the interaction with *S*. *scitamineum*. The reference set of sugarcane unigenes consisted of 72,268 sequences obtained from a de novo RNAseq assembly and a transcriptome annotation for six cultivars collected in various sugarcane crop fields, including the ‘RB925345’ variety [29]. This set of unigenes was used to allow cross-comparisons among the sugarcane sequencing data. To define the best alignment of the RNAseq to the unigenes set we performed two analyses. Using both Burrows-Wheeler Aligner (BWA) [31] and Bowtie2 [30] softwares, approximately 73% of the high-quality sequence reads were aligned to 67% of the unigenes (S3 File). The remaining subset of reads (15,000,000 PEs), those that showed no similarity to the sugarcane unigenes, was clustered using Trinity v2.0.6 [32]. A total of 25,794 contigs of more than 500 bp was assembled, and 16,219 were defined as ‘RB925345’ transcripts based on the presence of orthologs in the Viridiplantae section of the UniProt database (UniProt release 2015_03) (S4 File). These transcripts were identified by the prefix “gg” and probably include those most related to the pathogen infection. This new set of transcripts (gg) was combined with the sugarcane unigenes [29] (88,487 transcripts) to define the final set of DEGs (S5 and S6 Files). Our goal was to assess differential gene expression that started shortly after inoculation (5-DAI) and continued through fungal sporogenesis and, subsequently whip emission (200-DAI). DEGs were defined for both 5-DAI and 200-DAI samples, and the intersection between the two transcript groups was used for the annotation processes (Fig 2).

It was not possible to detect DEGs using FDR in 5-DAI samples. However, we pursued the analysis cautiously using p-values set at less than 0.05 and 0.01. The subtle sugarcane response shortly after inoculation may be due to the low percentage of the fungus in buds and/or delayed plant response due to the susceptibility of the variety used in the experiment [25]. Only 2% of the total reads were detected as genes expressed by the fungus, which represents approximately 67% of the *S*. *scitamineum* complete set of genes [26].

### Enrichment Analysis of GO Terms

**Fig 3:**
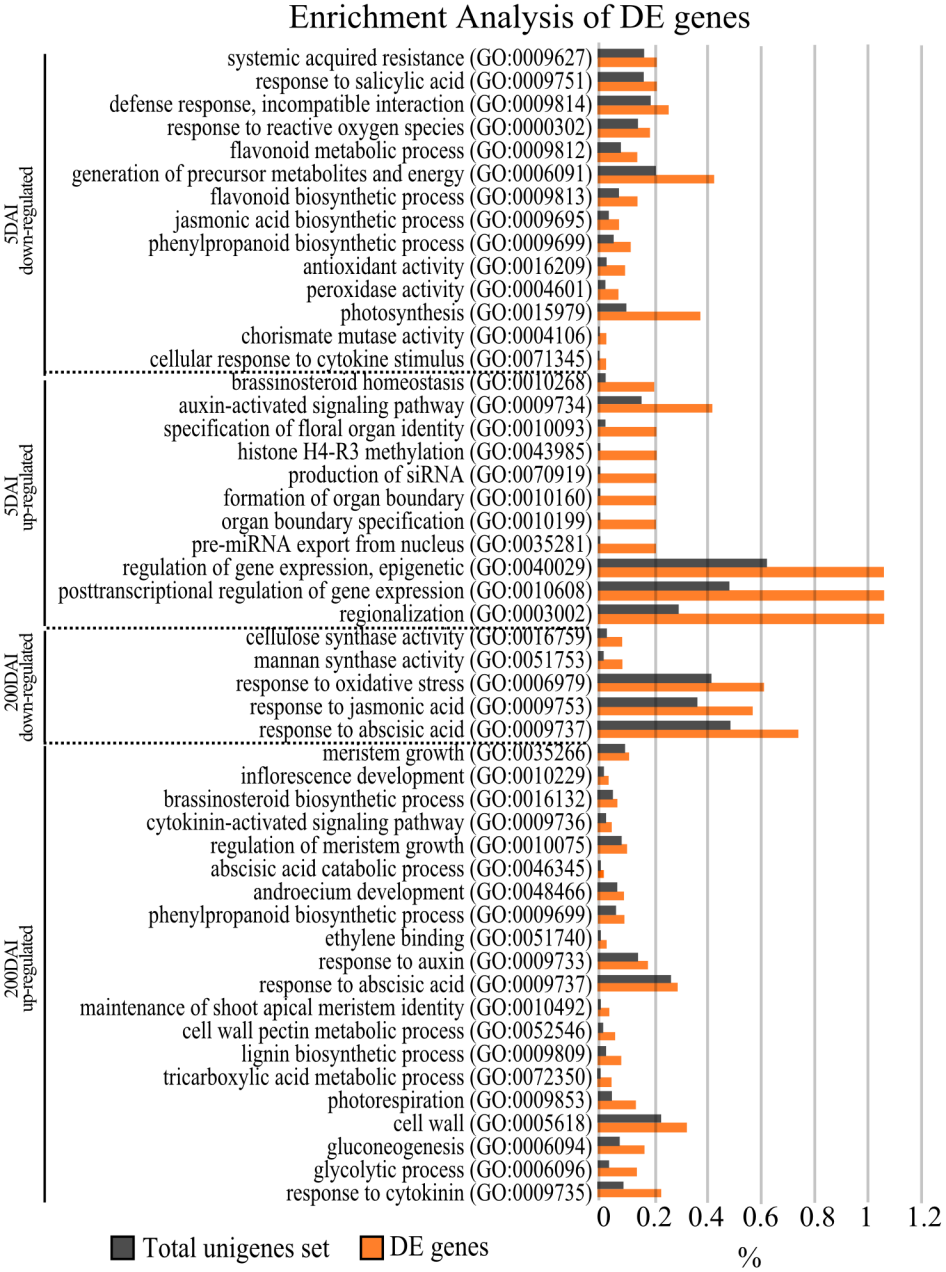
Enrichment analysis of GO terms. DEGs were submitted to enrichment analysis in BLAST2GO software, and a p-value ≤ 0.05 was used as the cut-off parameter. The gray bars represent the percentage of genes related to each selected GO term in the total set of sugarcane unigenes. The red bars represent the percentage of genes related to each selected GO term in the set of DEGs. The complete list of enriched GO terms in each set of DEGs can be found in S6 File.

The molecular events underlying sugarcane response during infection were initially suggested based on GO terms assignment and GO enrichment analysis of DEGs (S7 File). The enrichment analysis of 5-DAI DEGs showed as expected two contrasting molecular responses as previously described [25]. The genes involved in general plant immunity were down-regulated, while those for epigenetic mechanisms were up-regulated (Fig 3). In addition, this same enrichment test included terms related to shoot apical activities with the identification of three GO terms: Regionalization, Organ boundary specification, and Specification of floral organ identity. These terms suggest that the plant meristem functions are prematurely modulated by the presence of the pathogen. Some genes related to this same functional group were also enriched after whip emission. The gene regulatory network for shoot apical functions known in plant models is responsible for the differentiation of cells and organs (leaves and inflorescences). In corn, smut fungi are known to prevent or modify floral organ differentiation inducing tumor-like galls [47,48]. We suggest that a similar modulation occurs in susceptible genotypes of sugarcane infected with *S*. *scitamineum* very *s*hortly after colonization. However, although the GO term enrichment test was in agreement with this hypothesis, it needs to be further investigated because 5-DAI DEGs were not supported by FDR. A time-course experiment using sugarcane varieties showing various levels of smut-related responses, for instance, should be conducted to determinate the expression profile of genes associated with the meristematic functions identified here.

**Fig 4:**
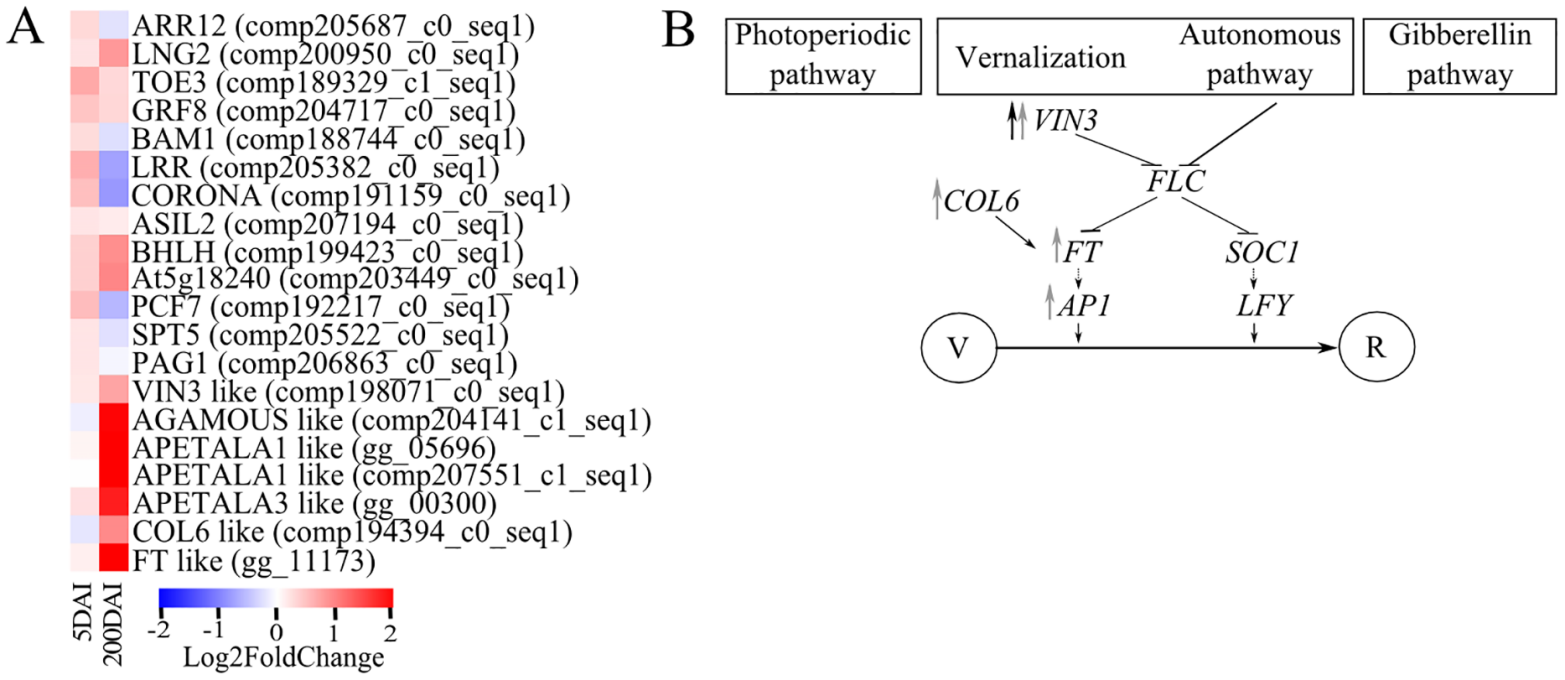
Sugarcane DEGs related to meristem functions. (**A)** Expression profile of genes related to meristem functions represented as values of a log2 fold change (inoculated/control). The heatmap was constructed using the R software package. Blue squares represent down-regulated genes, and red squares represent up-regulated ones. The statistical significance of expression regulation is presented in S5 and S6 Files. (**B)** Model of probable events related to whip development in sugarcane. Increase in VIN3 expression early in infected plants may release FT expression, which in turn positively regulates Apetala-1 (AP1) expression, turning the vegetative growth program to reproductive, via the autonomous/vernalization pathway. The black arrow represents up-regulation at 5 DAI, and the gray arrows represent up-regulation at 200 DAI.

**Fig 5:**
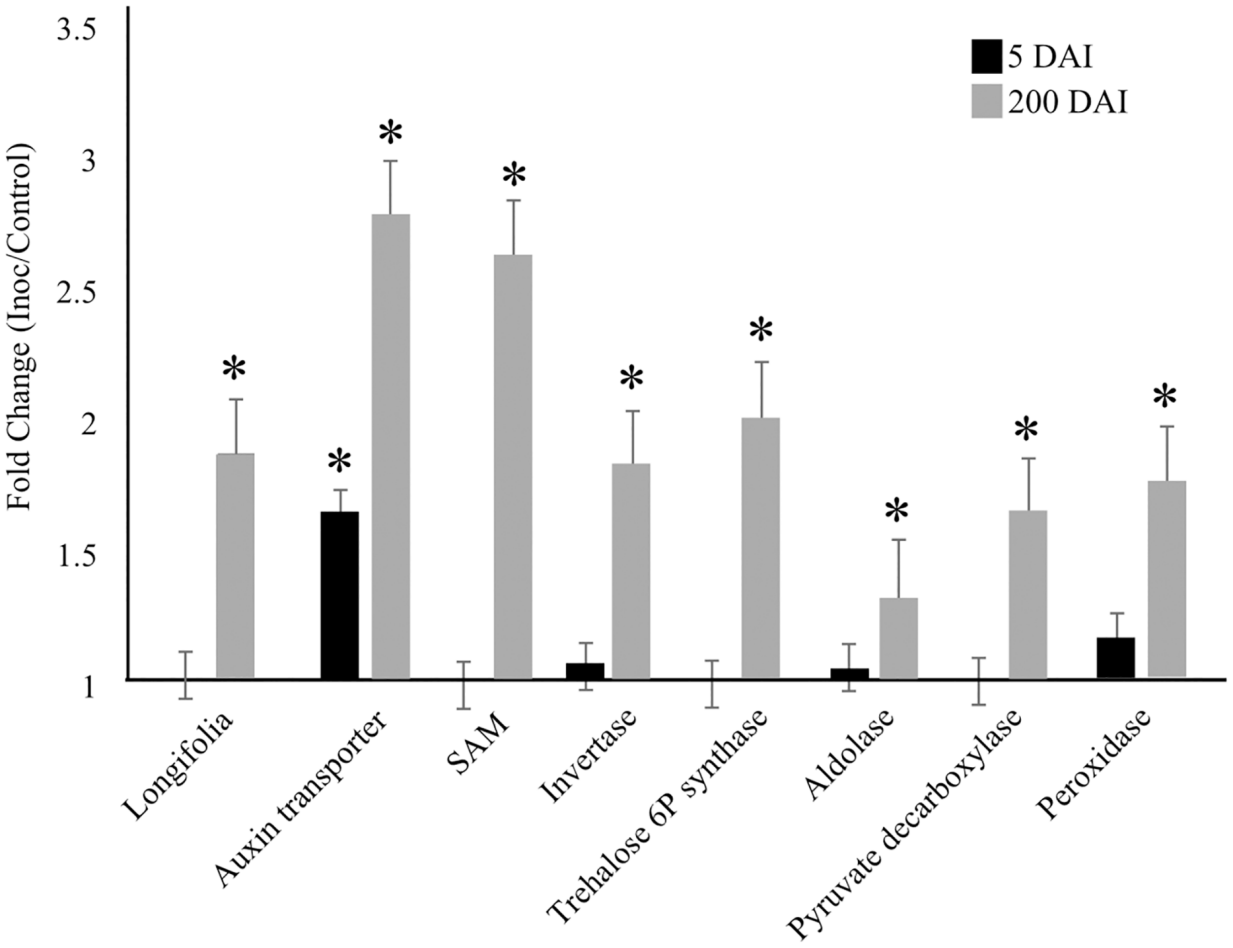
RT-qPCR validation. Sugarcane unigenes selected for RT-qPCR analysis of 5-DAI and 200-DAI samples: longifolia-like protein (*comp200950_c0_seq1*); auxin transporter (*comp205699_c0_seq1*); SAM (*comp194455_c0_seq1*); invertase (*comp201528_c0_seq1*); trehalose 6P synthase (*comp204716_c0_seq1*); aldolase (*comp196354_c1_seq1*); pyruvate decarboxylase (*comp200606_c0_seq1*) andperoxidase (*comp187834_c0_seq1*). The reactions were performed using a one-step GoTaq^®^ One-Step RT-qPCR System Kit (Promega) using a 7500 Fast Real-Time PCR System (Applied Biosystems). Statistical analysis was performed using REST^®^ software. “*” indicates genes differentially expressed in the RT-qPCR reactions (p-value < 0.05).

The meristem-related functions were remarkably affected considering the enrichment test of200-DAI sample DEGs (S7 File). Several identified genes allowed us to propose a putative model (Fig 4) for the transition of the normal meristematic functions controlled by the interaction of auxin and cytokinin hormones [49] to the development of the whip. For instance, a *longifolia-like* gene (LNG, *comp200950_c0_seq1*) is up regulated at this time point. Mutants overexpressing this same gene in *A*. *thaliana* have long petioles, narrow but extremely long leaf blades with serrated margins, elongated floral organs, and elongated siliques as a result of polar-cell elongation [50]. This description resembles the whip development in sugarcane. The LNG-like gene was assayed by qPCR, confirming the RNAseq data (Fig 5). Transcripts encoding the homologs of VIN3 (*vernalization insensitive 3 protein*) were also up-regulated after whip emission. VIN3-like proteins are involved in both the vernalization and photoperiod pathways by regulating the expression of the floral repressors FLOWERING LOCUS C (FLC) and FLOWERING LOCUS M (FLM). In *A*. *thaliana*, the VIN3-LIKE protein epigenetically represses a member of the FLC family, enabling flowering in the non-inductive photoperiods [51]. Additional transcripts members of three gene classes responsible for floral development (A, B and C) were identified. The ABCDE model proposes that a certain combination of MADS proteins activates different groups of genes related to flower [52,53]. A MADS-box TF homologous to AP1 (APETALA1; class A) was detected to be highly expressed (*comp207551_c1_seq1*; log_2_ fold-change = 9.4). This gene is essential in *A*. *thaliana* for the transition from an inflorescence meristem to a floral meristem [54]. This same MADS-box TF, along with the product of FLOWERING LOCUS T (FT; *gg_11173*), which is also highly expressed in infected sugarcane plants (log_2_ fold-change = 2.74), promotes the transition from vegetative to reproductive growth. In *A*. *thaliana*, FT encodes a small peptide that is recognized as the major component of florigen, which induces the expression of other floral genes, such asAP1 [55]. Three other MADS-box TFs were up-regulated after whip emission, encoding the homologs of APETALA3 (AP3, *gg_00300*), the class B gene AGAMOUS (AG, *comp204141_c1_seq1*), the class C gene APETALA1(AP1, *gg_05696*) and COL6 (C2C2-CO-like transcription factor; *comp194394_c0_seq1*). COL6 belongs to the CONSTANS family and encodes a putative zinc finger TF that promotes the induction of flowering in *A*. *thaliana* during long photoperiods [56] through activation of floral meristem-identity genes, such as LEAFY [57]. Regulatory switches coordinating these developmental changes have been extensively studied in *A*. *thaliana* [54]; theyare very precise and may vary in sugarcane, but the enrichment of genes related to the transition in meristem functions led us to associate these events with the plant-pathogen interaction mode [54]. It seems reasonable to assume that a combination of MADS-box TFs that are up-regulated in smut-infected plants may coordinate the gene expression related to whip development as an alternative route instead of the normal flowering program.

Given the interaction of *S*. *reilianum* with maize [48] and the data presented here, both *Sporisorium* species may share a common trend of modifying the meristem identity. In maize, phyllody and tumor formation result from alterations in the floral developmental program at both the apex and axillary meristems [48]. In sugarcane, the whip may result from releasing the transition from vegetative to reproductive/flowering, potentially via the autonomous/vernalization pathway [51] (Fig 4B).

### Hormonal Imbalance Plays a Role in Sugarcane Smut Disease

Changes in the expression profiles of genes related to the regulation, synthesis, and transport of hormones identified in the enrichment analysis were investigated. We detected that JA (jasmonic acid)-mediated as well as SA (salicylic acid) signaling are potentially restrained in the experiments at 5-DAI (Fig 3). Indeed Que et al. [25],using a smut-resistant sugarcane genotype, identifiedthe overexpression of JA-associated genes.

**Fig 6:**
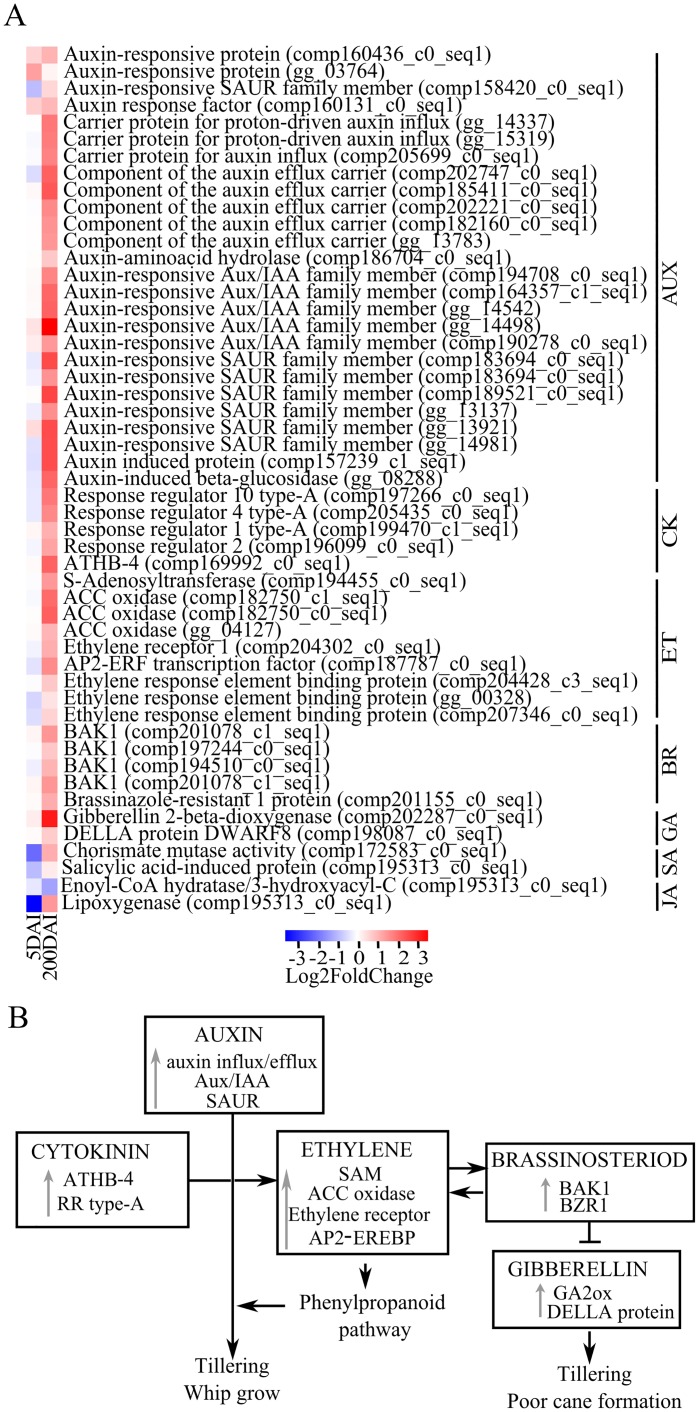
DEGs related to hormone biosynthesis and signalization. **(A)** Heatmap constructed using the R software package and log2 fold change values (inoculated/control). The left column represents the regulation of each gene at 5 DAI, and the right column represents the regulation of each gene at 200 DAI. **(B)** Model concerning the contribution of the main plant hormones with up-regulated DEGs in sugarcane after whip emission.

In contrast to SA and JA, auxin-activated signaling pathways are up-regulated. In addition to acting as a negative regulator of the plant immune system [58, 59], we suspect that auxin-related DEGs are associated with the meristem transcriptional reprogramming during whip emission because an increased auxin transporter gene expression was confirmed by RT-qPCR in both 5- and 200-DAI samples (Fig 5). The balance of auxin-cytokinin is reported to be essential for typical meristem function [49]. Additionally, auxin is the hormone with the highest number of responsive genes up-regulated after whip emission (Fig 6 and S8 File), including those involved in auxin influx/efflux, auxin-amino acid hydrolase, and auxin-responsive proteins, such as Aux/IAA, SAUR, and auxin-induced β-glucosidase. In the *S*. *reilianum*-maize pathosystem, the floral reversion process is partially attributed to an increase in auxin concentration, contributing to the loss of apical dominance and a greater number of ears per branch [48]. Auxin-dependent signaling is likely necessary for whip emission, which also involves the loss of apical dominance and growth of secondary buds [3]. Anincrease in auxin-like substances wasreported by Hector et al. [60]using smutted sugarcane extracts. The authors suggested that the balance between auxin and cytokinin is disrupted in infected sugarcane plants.

Although cytokinin is a hormone that is often related to tillering [61], a symptom often related to smut, genes related to its synthesis were not detected among those that were differentially expressed (Fig 6). However, several type-A response regulators (RRs) were up-regulated at 200 DAI. Type-A RRs negatively regulate cytokinin signaling by repressing type-B RRs, and they are transcriptionally up-regulated in response to cytokinin [62]. Cytokinins are central regulators in maize smut caused by *U*. *maydis*. The fungus can synthesize cytokinins, an important virulence factor that is associated with uncoordinated cell division and tumor formation [63,64]. The *S*. *scitamineum* genome SSC39B [26] does not have cytokinin biosynthetic gene homologs. However, previously data have shown that *S*. *scitamineum* secretes some cyokinin-like substances that can potentially activate cytokinin-responsive genes [65].

Regarding ethylene (ET), DEGs related to biosynthesis, perception, and signal transduction were detected after whip emission (Fig 6). For instance, SAM (S-adenosyl-L-methionine) was identified as being up-regulated, which was confirmed by RT-qPCR analysis (Fig 5). ET is often related to the lignification of plant tissues by increasing the expression of genes involved in the phenylpropanoid pathway [66,67].

The genes related to brassinosteroids were also up-regulated. They included several BAK1 LRRs [68] and the transcription repressor BZR1, which binds directly to the promoters of feedback-regulated brassinosteroid biosynthetic genes [69]. The identification of GA2ox and DELLA proteins among the DEGs suggests a blockage of GA signaling because the products of these genes act by reducing the availability of active GAs and repressing GA-responsive genes, respectively [70,71] (Fig 6). For instance, rice plants overexpressing C20-GA2ox exhibit early tillering, adventitious root growth, and changes in plant architecture that generate semi-dwarfs [70]. Diseased sugarcane plants have reduced node distance and poor cane formation ([3], our own observations), similar to the symptoms described in rice with low GA levels.

### Carbon Distribution Is Affected by *S*. *scitamineum* Colonization

**Fig 7:**
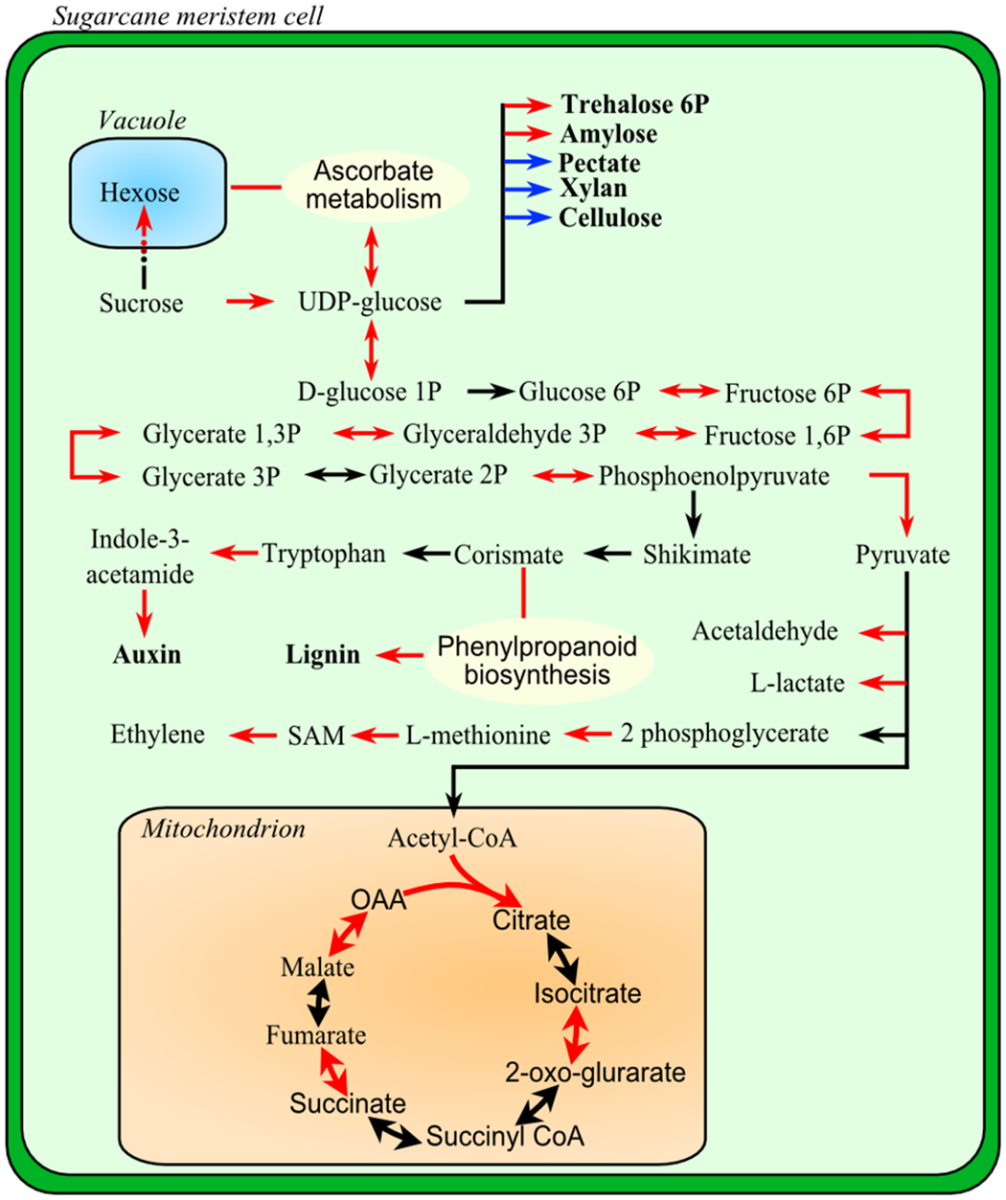
Smutted sugarcane metabolism in the late moments of interaction. Schematic representation of smutted sugarcane metabolism in the whip base (200 DAI). Red and blue arrows represent up- and down-regulated expression, respectively, and black arrows are unchanged expression.

In biotrophic interactions pathogen growth relies on host nutrients derived from active metabolism. Sucrose and its derivatives are central molecules involved in carbohydrate translocation, metabolism, and sensing in higher plants [72]. Invertases were among the DEGs up-regulated after whip emission and included a neutral alkaline invertase (*comp189016_c0_seq1*) and soluble acid invertase (*comp201528_c0_seq1*), both of which were confirmed by RT-qPCR analysis. Invertases catalyze the irreversible hydrolysis of sucrose (EC 3.2.1.26), in some cases leading to a shift of the apoplastic sucrose/hexose ratio in favor of hexoses [73], which regulate many aspects of plant metabolism such as carbohydrate portioning, developmental processes, and hormonal responses to biotic stress [73,74]. Plant invertases are classified into three groups: alkaline/neutral invertases localized in the cytosol, mitochondria or plastids and two types of acid invertases, one insoluble and bound to the cell wall (cell wall invertase CWI) and the other soluble in the vacuole space (vacuolar invertase VI) [73,74]. The acid invertases CWI play a role in sucrose partitioning, plant development and cell differentiation, whereas the VIs determine the sucrose level stored in the vacuole and its remobilization for metabolic processes. The up-regulation of vacuolar and neutral invertases represents a shift in the plant’s metabolism that targets carbon to pathways unrelated to sucrose storage, which can aggravate sugarcane smut symptoms. Additionally, it has relevant implications for the hexose-based sugar signaling system involved in plant immunity [75]. Increased hexose levels can also be related to the nutrients supplied to the pathogen during teliospore differentiation and whip formation (Fig 7). The importance of the sugar content in signaling for axillary bud growth was recently demonstrated and indicates that, in addition to auxin, an increased sugar supply is necessary and sufficient for suppressed buds to be released from apical dominance [76]. Redistribution of the host carbon in response to *S*. *scitamineum* sporogenesis is suggested by the transcriptional profiles of the genes related to glycolysis, the citric acid cycle, sucrose, starch, xylan, trehalose 6P, and cellulose biosynthesis (Fig 7), findings that were also confirmed by RT-qPCR analysis (Fig 5). It has been suggested that, rather than playing a metabolic role, the low concentration of trehalose-6P (T6P) in infected plants functions as a regulatory component. Trehalose-6P synthase can sense sucrose availability to generate T6P as a signal to promote growth [72,77,78]. There is also evidence in *A*. *thaliana* that T6P acts as an endogenous signal to control the transition from vegetative growth to flowering by increasing trehalose-6P synthase transcript levels [72,79]. These findings should encourage new experiments to better understand the sugarcane metabolic response to smut as the disease progresses, the turning point at which the plant changes its metabolism to allow fungal sporogenesis, and the significance of this shift to teliospore/whip differentiation.

Several transcripts related to lignin biosynthesis (Fig 5) were detected as being up-regulated after whip emission such as shikimate hydroxycinnamoyl transferase (EC 2.3.1.133), cinnamoyl-CoA reductase (EC 1.2.1.44), and peroxidase (EC 1.11.1.7). In addition, up-regulated laccases were also detected, such as DEGs (*gg_01080*, *gg_14238*, *gg_10439*, *gg_15488*). Plant laccases (EC 1.10.3.2) are glycoproteins involved in lignin biosynthesis through the oxidation of lignin precursors [80]. An increase in the lignification of smut-resistant plants has been detected by measuring cinnamyl alcohol dehydrogenase levels and by the overexpression of genes in RNAseq experiments of resistant varieties in the early moments of interaction [25,81]. The increase in lignin content after whip emission is probably not related to a protective host response; instead, it is likely a stage in the formation of the whip, which is composed of lignified plant tissue [82]. Recently, a proteomic approach developed after whip emission revealed 53 proteins related to lignin accumulation and oxidative stress at this stage of disease symptoms [83]. Responses regarding ROS (*Reactive Oxygen Species*) in 200-DAI samples were also detected at the RNA level. Nineteen DEs were identified as being related to ROS-scavenging enzymes (S9 File), including 16 up-regulated DEGs (one catalase, eight peroxidases, two thioredoxins, and five glutathione S-transferases) and three down-regulated DEGs (two peroxidases and one thioredoxin). These results suggest that the ROS level is high in *S*. *scitamineum*-colonized cells during sporogenesis.

### Sequence Features of Resistance Gene Analogs (RGAs) Differentially Expressed

**Fig 8:**
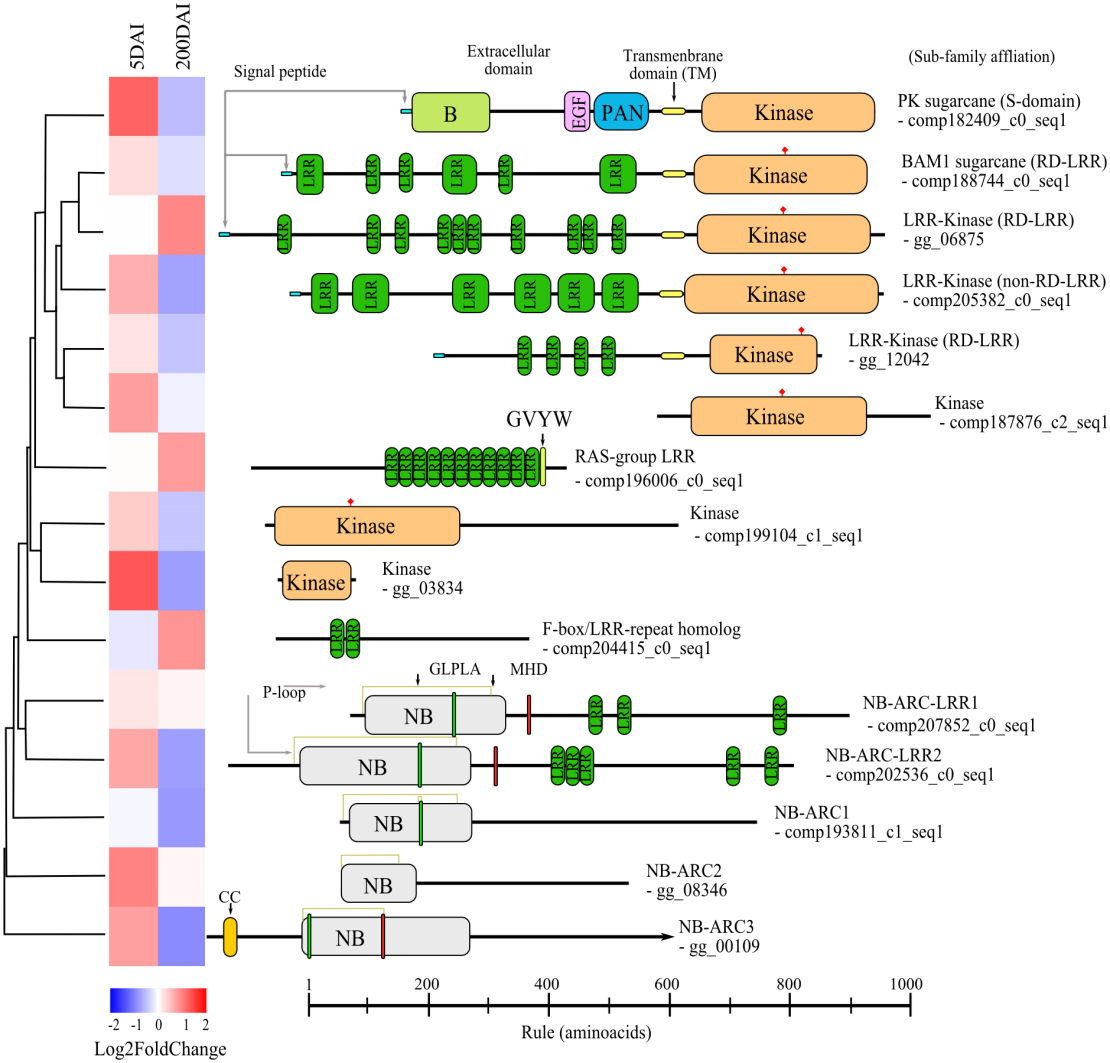
Resistance gene analogs (RGAs) detected as DEGs in smut-infected sugarcane in early and late interaction. The guide tree was obtained based on translated amino acid sequence similarity using CLUSTALW2. The heatmap represents the respective expression log2 fold change values. Protein structural features: signal peptide sequences predicted by SignalP 4.1 (http://www.cbs.dtu.dk/services/SignalP/); transmembrane domains predicted by TMHMM Server v. 2.0 (http://www.cbs.dtu.dk/services/TMHMM/). Other domains, motifs and active sites were predicted by InterProScan. CC, coiled coil; kinase (IPR011009); NB, nucleotide-binding (IPR002182); B, Bulb-type lectin domain (IPR001480); EGF, epidermal growth factor domain (IPR000742); PAN domain (IPR03609); LRR, leucine-rich-repeat (IPR001611; IPR003591);red pins represent kinase active sites, and gray arrows are P-loops of N-terminal NB-ARC proteins.

Resistance gene analogs (RGAs) were analyzed for both time points. The predicted domains and other sequence features that are potentially important for RGAs function were identified (Fig 8). Although this study was conducted with an intermediate-resistant genotype, we detected promising candidates associated with this particular biotrophic interaction and their potential role in the disease progression mechanism proposed here. Several RGAs containing leucine-rich repeat (LRR) domains have already been identified in sugarcane [84]. Three of them were also found here: the two BAM-related proteins *comp_188744* and *gg_06875*(RGA482) and a protein encoded by *comp_187876* (RGA367) (Fig 8). All of the other proteins predicted in this work are new discoveries in sugarcane.

BAM orthologs are leucine-rich repeat receptor-like serine/threonine-RD kinases (LRR-RLKs) [85] and receptors for signals to switch meristem identity. BAM proteins play roles equivalent to those of CLV (**CL**A**V**ATA) proteins in *A*. *thaliana*, representing a functional redundancy within the program related to meristem functionality [86]. Other LRR-RLKs, such as ERECTA, were first described in relation to plant development, and only later were they positively associated with disease resistance [85].

Other kinases were identified sharing amino acid identities with the RLKs of different families (Fig 8). For instance, *comp205382_c0_seq1* encodes a transmembrane protein (non-RD kinase) harboring an LRR domain [85]. The translated amino acid sequence is most similar to the protein Xa21 in *O*. *sativa*, sharing all conserved residues [87]. The *xa21* gene is known to confer resistance to *Xanthomonas oryzae* pv. *oryzae* race 6 in rice [87]. LRR-RLK-harboring domains shared by plant S-locus glycoproteins and S-receptor kinases (SRK) (IPR003609) were also detected (*comp182409_c0_seq1*). SRKs were first described as being associated with *Brassica* self-incompatibility, but were later related to the perception of pathogen infection, probably by binding to a glycoprotein inducer, such as cellulose or chitin [88]. A transcript (EST) similar to *comp182409_c0_seq1* was detected by BLAST (GenBank) in *Oryza longistaminata*. *Comp182409_c0_seq1* shares an 87% identity to the 3′ end of the *xa21* nucleotide sequence (FF359116), which is the portion coding for the kinase domain. No sugarcane ESTs similar to *comp182409_c0_seq1* were found in the NCBI-expressed sequence tags database.

*S*. *scitamineum* also induced the presence of cytoplasmic LRR proteins known as plant intracellular *Ras* group-related LRR proteins (PIRLs) [89]. The protein encoded by the *comp196006_c0_seq1* DEG at 200 DAI is most similar to other PIRL4s and contains the conserved GxxxVxxYxxxxW (‘GVYW’) motif immediately following the LRR domain.

The expression of an F-box/LRR-related gene was also detected in 200-DAI samples. F-box proteins are part of the SCF (SKP1/Cullin/F-box) ubiquitin ligase complex involved in protein degradation (proteasome) [90]. In *O*. *sativa*, theF-box/LRR-repeat MAX2-homolog controls tillering by suppressing axillary bud activity, potentially by degrading specific proteins that activate axillary growth [91]. The *comp204415_c0_seq1* DEG encoded a protein that has an 83% amino acid identity with the rice MAX2-homolog (Q5VMP0). Tillering is one of the earlier disease symptoms related to smut [3].

Cytoplasmic proteins attached to the nucleotide binding-ARC (NB-ARC) domains and containing an ATPase and a nucleotide-binding site [92,93] compose the last class of LRR proteins identified among the translated transcripts that were differentially accumulated. The translated protein sequence of *comp202536_c0_seq1* contains the conserved GLPLA and MHD motifs that are essential to the function of other resistance proteins [93]; although lacking an obvious CC-domain at the N-terminus, this gene probably belongs to one of the CC-domain-containing subfamilies. Its sequence is most similar to an *O*. *sativa* gene assigned to chromosome 4 (CAE03396). All of these RGA-like encoding proteins are potential targets for functional characterization as receptors of signals due to the presence of *S*. *scitamineum*.

## Conclusion

This work reveals transcriptional changes associated with the most characteristic symptom of sugarcane smut disease. We speculate that whip emission is a consequence of premature transcriptional changes in meristem function (5-DAI) that results in the restraint of floral development via the vernalization pathway by increasing VIN3, COL6, FT, and AP1 gene expression and other flowering-related transcriptional factors (200-DAI). Fungal sporogenesis and whip emission are most related to auxin mobilization followed by a strong response of ROS-scavenging enzymes. In addition, the role of other plant hormones is also suggested. Because the processes associated with fungal development and whip emission require energy, the carbon partitioning of sugarcane is the most affected. The gene expression profile indicates that smutted sugarcane metabolism shifts towards energy production, increasing the expression of genes involved in glycolysis and TCA. The synthesis of signalizing molecules, such as trehalose 6P, is also among the results described. Increased expression of genes involved in lignin biosynthesis and sucrose breakdown are potential markers of whip development. We also described the RGA expression patterns involved in this particular interaction, leading to effective fungal colonization and disease establishment. *S*. *scitamineum* is known to colonize not only susceptible plants but also smut-resistant genotypes that, in response to unknown signals, allow unexpected fungal sporogenesis and whip emission. This detailed work is an attempt to expose molecular mechanisms and candidate genes that can possibly reveal ways to control sugarcane smut disease.

### Database Accession Number

The sequencing data has been deposited at DDBJ/EMBL/GenBank under the BioProject ID PRJNA291816.

## Supporting Information

S1 File **A)**Experimental design used to produce biological insights of the interaction sugarcane-*S*. *scitamineum*.**B)** Number of whips developed each month after smut inoculation in the intermediate resistant genotype RB925345. **C)** Amplicons of primers Hs and Ha using total DNA of buds collected 5 DAI.
(PDF)

S2 File Primers used for qPCR validation of RNAseq analysis. (XLS)

S3 File **A)** Total of RNAseq data obtained and mapping results of BWA and Bowtie2 softwares against sugarcane unigenes. **B)** Percentage of reads mapped in *S*. *scitamineum* SSC39B genome.
(XLSX)

S4 File Fasta file of the new sugarcane transcripts assembled (gg). (TXT)

S5 File **A)** Mapping results and differential expression analysis using as reference sugarcane unigenes. **B)** IDS of sugarcane unigenes differentially expressed during interaction with *S*. *scitamineum* in early and late moments.
(XLSX)

S6 File **A)** Mapping results and differential expression analysis considering the new sugarcane transcripts (gg) obtained from non-mapped reads in sugarcane unigenes. **B)** IDS of new sugarcane transcripts differentially expressed during interaction with *S*. *scitamineum* in early and late moments. **C)** Blast2GO annotation of new sugarcane transcripts differentially expressed during interaction with *S*. *scitamineum* in early and late moments.
(XLS)

S7 File GO terms enrichment analysis of sugarcane. **A)** down-regulated DEGs at 5 DAI. **B)** GO terms enrichment analysis sugarcane up-regulated DEGs 5 DAI. **C)** GO terms enrichment analysis of sugarcane down-regulated DEGs 200 DAI. **D)** GO terms enrichment analysis of sugarcane up-regulated DEGs 200 DAI. **E)** GO terms enrichment analysis sugarcane down-regulated DEGs (gg) 5 DAI. **F)** GO terms enrichment analysis of sugarcane up-regulated DEGs (gg) 5 DAI. **G)** GO terms enrichment analysis of sugarcane down-regulated DEGs (gg) 200 DAI. **H)** GO terms enrichment analysis of sugarcane up-regulated DEGs (gg) 200 DAI.
(XLS)

S8 File DEGs hormone-related at 200 DAI. (XLS)

S9 File DEGs ROS-related at 5 DAI and 200 DAI. (XLS)
