# Supplementary material for: RNAseq Transcriptional Profiling following Whip Development in Sugarcane Smut Disease
Source: PLoS One. 2016 Sep 1;11(9):e0162237. doi: 10.1371/journal.pone.0162237 (PMC5008620; doi:10.1371/journal.pone.0162237)
Supplement: S1 File — A)Experimental design used to produce biological insights of the interaction sugarcane-S. scitamineum.B) Number of whips developed each month after smut inoculation in the intermediate resistant genotype RB925345. C) Amplicons of primers Hs and Ha using total DNA of buds collected 5 DAI. (PDF) [file pone.0162237.s001.pdf]

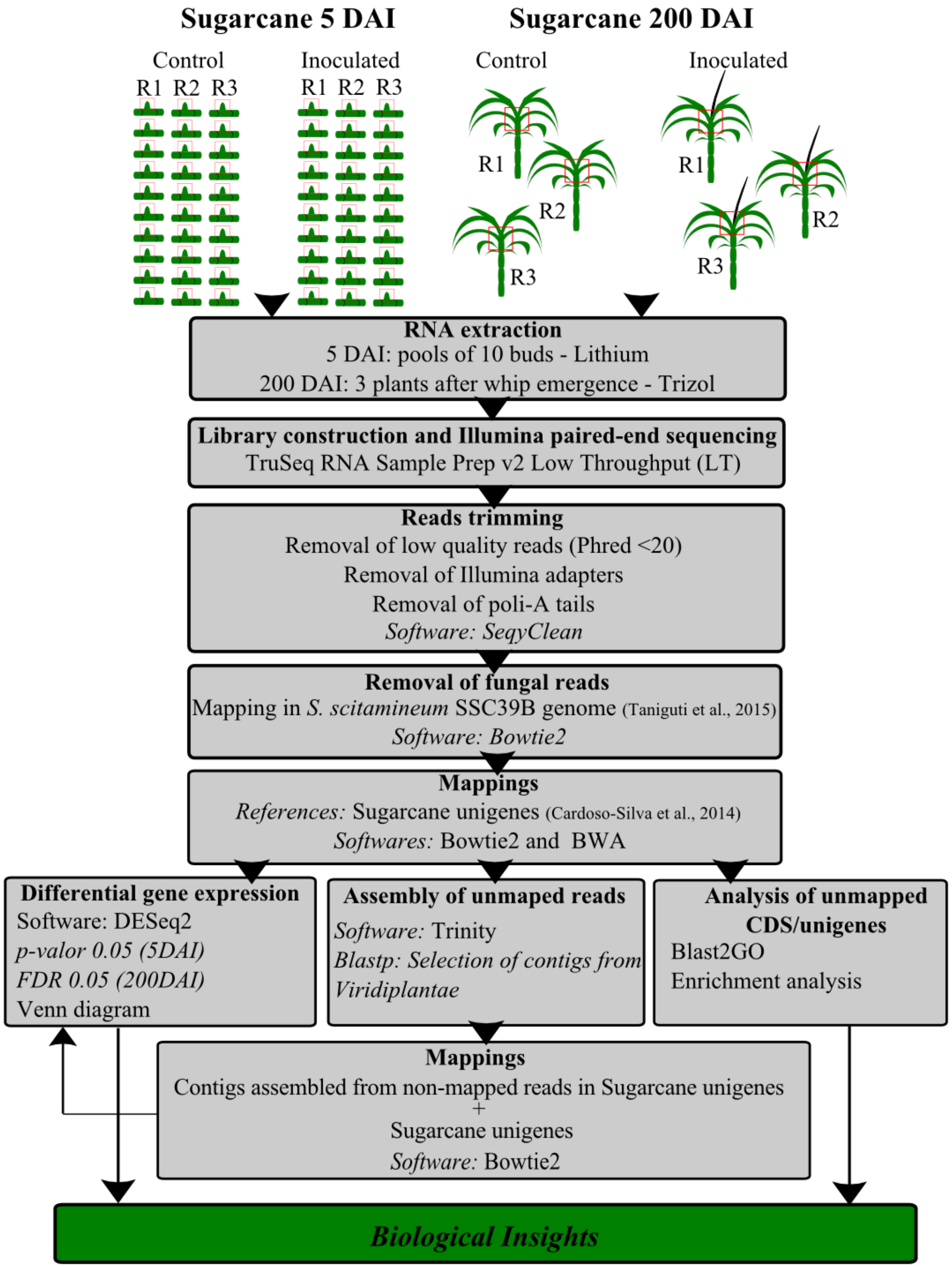

Fig A. Experimental design to investigate transcriptional changes of sugarcane plants of the intermediate resistant variety "RB925345" in response to *S. scitamineum* development. The time points analyzed were: 1) 5 DAI (days after inoculation); and 2) 200 DAI after whip emission. Single budded setts of seven month-old plants were surface disinfected, heat treated (52°C for 30 min in water bath, 1 kg of buds/6L of water) and incubated for 16 h at 28°C. Artificial inoculation was performed using the paste method in previously needle damaged buds to overcome mechanical/pre-formed resistance. Mock inoculated plants were used as control. A greenhouse experiment was conducted in completely randomized design with two treatments (5 DAI and 200 DAI) and three replications: 1) three pools of 10 breaking buds were used to determinate transcriptional changes five days post-mock or inoculation with *S. scitamineum;* and 2) three diseased plants after whip emission and three healthy plants of the same age were used to determinate transcriptional changes 200 DAI. RNA extraction methods used were: 1) 5 DAI, lithium-based protocol (Gasic et al., 2004); and 2) 200 DAI, TRIzol® (Life Technologies #15596-018) according to manufacturer's instructions.

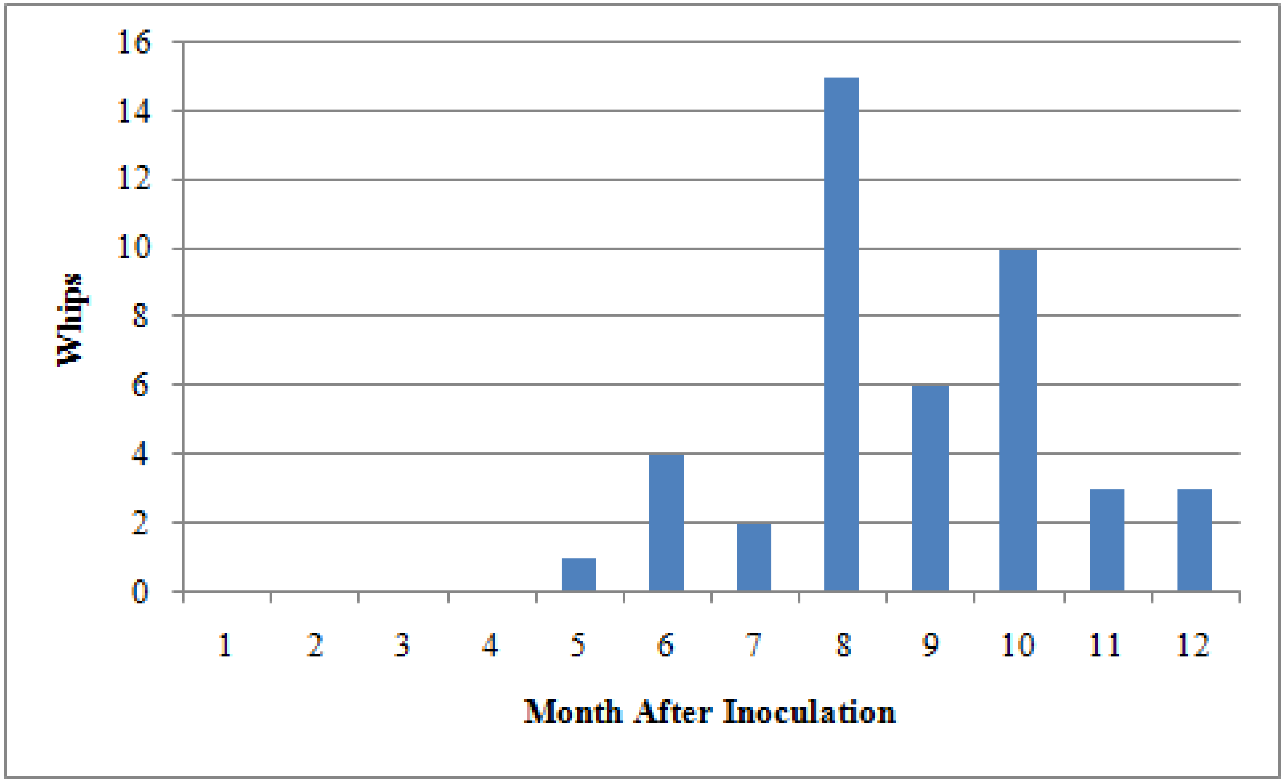

Fig B. Number of whips developed each month after smut inoculation in the intermediate resistant genotype RB925345.

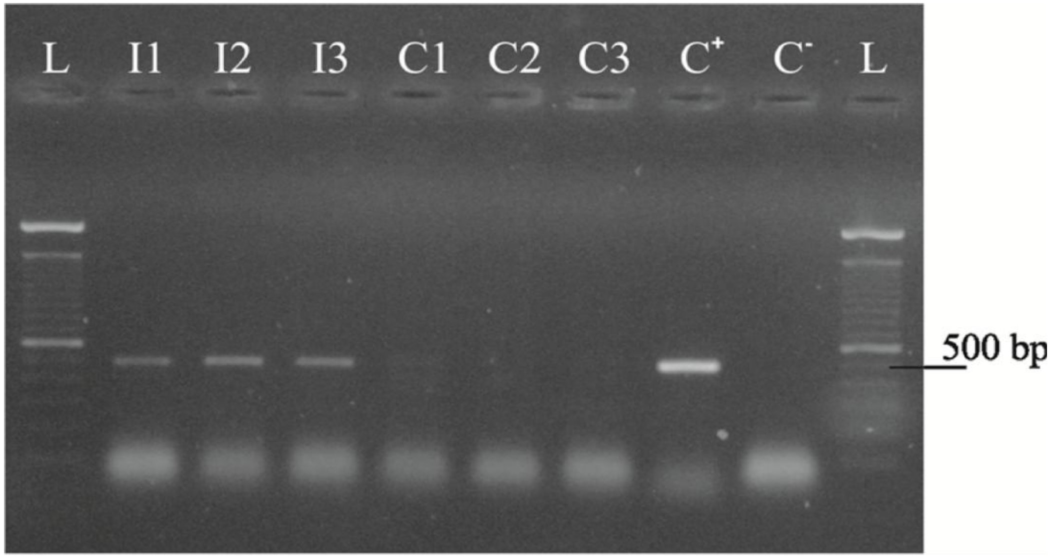

Fig C. Amplicons of primers Hs and Ha using total DNA of buds collected 5 DAI.
